# Attention and word learning in autistic, language delayed and typically developing children

**DOI:** 10.3389/fpsyg.2014.00490

**Published:** 2014-05-26

**Authors:** Elena J. Tenenbaum, Dima Amso, Beau Abar, Stephen J. Sheinkopf

**Affiliations:** ^1^Brown Center for the Study of Children at Risk, Women and Infants HospitalProvidence, RI, USA; ^2^Department of Cognitive, Linguistic and Psychological Sciences, Brown UniversityProvidence, RI, USA; ^3^School of Medicine and Dentistry, University of Rochester Medical CenterRochester, NY, USA; ^4^Department of Psychiatry and Human Behavior, Brown UniversityProvidence, RI, USA

**Keywords:** autism spectrum disorders, eye tracking, word learning, attention to faces, language development

## Abstract

Previous work has demonstrated that patterns of social attention hold predictive value for language development in typically developing infants. The goal of this research was to explore how patterns of attention in autistic, language delayed, and typically developing children relate to early word learning and language abilities. We tracked patterns of eye movements to faces and objects while children watched videos of a woman teaching them a series of new words. Subsequent test trials measured participants‘ recognition of these novel word-object pairings. Results indicated that greater attention to the speaker‘s mouth was related to higher scores on standardized measures of language development for autistic and typically developing children (but not for language delayed children). This effect was mediated by age for typically developing, but not autistic children. When effects of age were controlled for, attention to the mouth among language delayed participants was negatively correlated with standardized measures of language learning. Attention to the speaker‘s mouth and eyes while she was teaching the new words was also predictive of faster recognition of those words among autistic children. These results suggest that language delays among children with autism may be driven in part by aberrant social attention, and that the mechanisms underlying these delays may differ from those in language delayed participants without autism.

## INTRODUCTION

Autism is a disorder marked by diminished attention to social information and is often associated with significant language impairment (American Psychiatric Association, 2013). Early word learning likely involves attention to social cues (Carpenter et al., 1998; Morales et al., 2000; Brooks and Meltzoff, 2005, 2008; Mundy et al., 2007), recognition of co-occurring items and labels (Smith and Yu, 2008; Smith et al., 2011; Trueswell et al., 2013), and certain biases or constraints regarding what things are likely to be labeled (Markman and Wachtel, 1988; Merriman and Bowman, 1989; Smith, 2000; de Marchena et al., 2011). While many potential word learning situations are quite ambiguous (Medina et al., 2011), the highly informative encounters, when they do occur, are likely to help bootstrap language learning. In these highly informative contexts, children have much to gain from attending to social cues.

For example, one way for a child to successfully encode a new label for an object would be to coordinate her attention with a speaker, interpret the speaker’s intent to label, isolate the label, and crucially, identify the referent for that label. Integration of this information would require attention to a number of social cues. Research supporting a social pragmatic account of word learning suggests that children who are sensitive to these social cues in infancy demonstrate superior language skills later in development (Carpenter et al., 1998; Morales et al., 2000; Brooks and Meltzoff, 2005, 2008; Mundy et al., 2007). An open question is whether language deficits in autism may be driven by the aberrant patterns of social attention associated with the disorder (Klin et al., 2002; Pelphrey et al., 2002; Chawarska and Shic, 2009; Shic et al., 2011; Chawarska et al., 2013; Jones and Klin, 2013). To address this question, we compared patterns of attention in a word-learning context between autistic children, children with language delays (but without the social deficits associated with autism), and language-matched typically developing children.

The most widely cited evidence for connections between social attention and word learning come from the joint attention literature. Typically developing infants begin responding to joint attention (RJA) between 6 and 12 months (Carpenter et al., 1998) and begin initiating joint attention (IJA) between 12 and 24 months (Carpenter et al., 1998; Bruinsma et al., 2004). Children with ASD, however, show impaired RJA and IJA throughout toddlerhood (Leekam et al., 1998; Dawson et al., 2004). This impairment has been described as a primary deficit and a root cause of many symptoms associated with ASD (Charman, 2003). Some claim that the lack of joint attention reflects muted interest in social interaction (Baron-Cohen, 1994) and that this lack of interest in turn prevents the child from gaining valuable information from other people (Mundy and Neal, 2000). In support of this possibility, the tendency to follow a speaker’s gaze to a target referent has been shown to predict language ability among typically developing children (Carpenter et al., 1998; Morales et al., 2000; Brooks and Meltzoff, 2005, 2008; Mundy et al., 2007) and children with ASD (Bono et al., 2004; Dawson et al., 2004). Recent work indicates that gaze following among autistic children is necessary but not sufficient for successful language learning and that recognition of the communicative relevance of gaze shifts are related to the autistic child’s level of social impairment (Gliga et al., 2012).

While following a speaker’s gaze to the target referent is clearly important for word learning, the speaker’s face also holds valuable communicative information. From the eyes, a child can gather information about who the speaker is addressing, what she may be referring to, and her affective response to the situation. By focusing on the speaker’s mouth, the child stands to gain multimodal information about the label being provided as well as information about how those sounds are produced. Typically developing infants focus a great deal of attention on a speaker’s mouth (Merin et al., 2007; Nakano et al., 2010; Frank et al., 2011; Lewkowicz and Hansen-Tift, 2012; Oakes and Ellis, 2012). Recent evidence suggests that infants who go on to develop autism also focus attention on the mouth, but diverge from typically developing infants in diminished attention to the eyes in late infancy (Jones and Klin, 2013). Attention the mouth seems to be adaptive for language learning in typical development; attention to the mouth in early infancy predicts larger vocabulary size in toddlerhood (Young et al., 2009).

Young et al. (2009) posited that attention to the mouth facilitates language development because infants who make use of visual information are better able to process the speech stream by relying on their already developed audiovisual integration. Given that audiovisual integration of speech may be atypical among children and adolescents with autism (Smith and Bennetto, 2007; Mongillo et al., 2008), it remains unclear whether attention to the mouth in this population would be similarly related to greater success in language development. Because the studies of audiovisual processing in autism have been done with older children and adolescents, it is not possible to determine whether inattention to audiovisual signals early in development lead to atypical audiovisual processing or if it is atypical audiovisual processing that leads to diminished attention to these cues. In either case, the potential contribution of attention to the mouth for language learning in autism remains unresolved.

Recent evidence from toddlers with ASD suggests that diminished attention to faces and to the mouth specifically is associated with atypical language profiles (Chawarska et al., 2012), but not with level of language ability. Evidence from teenagers with ASD suggests that increased attention to the mouth is related to greater communicative competence among verbal and language impaired participants (Norbury et al., 2009).

While the stimuli used in these studies were social, they were not specific to processes of word learning. Because we were interested in the use of information in the face for the purposes of word learning, we used eye tracking to monitor fixation patterns as children watched video presentations of a word-learning task adapted from Baldwin (1991). Children saw videos of an experimenter labeling one of two novel objects (“Wow, it’s a toma!”). At test, children saw the two objects again and were asked to, “Look at the toma!” Attentional distribution on familiarization trials was then analyzed with respect to the child’s success at encoding the novel object-label pair and language abilities on a standardized measure of language development.

This study was designed to explore how social attention supports language learning at the mechanistic level both within the word learning encounter and for general language development. We therefore focused on participants at the early word-learning stages of development. Typically developing participants were language matched to the children with ASD. This resulted in a typically developing sample that was significantly younger than the autistic participants. Language delayed participants were included to provide a control group who were closer in age to the ASD children (and roughly language matched).

We had four main hypotheses. First, based on previous findings using video stimuli in which a speaker directly addresses a child (Chawarska et al., 2012), we predicted that autistic children would look less at the stimuli and less at the speaker’s face and mouth than the control groups. Second, based on previous findings with typically developing and at-risk children, we predicted that attention to the mouth would be related to better language skills among typically developing children (Young et al., 2009). Third, based on a view that language deficits in autism stem from atypical gaze which results in missed opportunities to learn from social scenes, we predicted that children with autism who do look at the informative areas of the scene for language learning (i.e., the eyes, mouth, and referent object) will be more successful at language learning than those who do not. Finally, we hypothesized that language impairments among those without autism are attributable to language specific impairments rather than social deficits, and thus we predicted that attention to social stimuli would be unrelated to language development among the language impaired participants without autism.

## MATERIALS AND METHODS

This experiment explored the distribution of attention in a word learning situation among three groups of children: typically developing (TD group), language delayed (LD group), and children with autism spectrum disorders (AD group). Participation involved two visits: a screening visit conducted in a developmental clinic and an eye tracking visit conducted in the laboratory. At the screening visit, diagnostic, cognitive, and language testing were administered and parents completed surveys. At the eye tracking visit, children’s fixations were monitored while they watched videos of a modified version of a word-learning paradigm used by Baldwin (1991). Order of the visits was based on the schedule of the family. Parents provided informed consent at the child’s first visit. Power analyses were run (Lenth, 2006-2009) in order to confirm that the current sample size was sufficient to test for a significant effect given effect size estimates for differences in attention to social stimuli obtained in previous studies (Chawarska et al., 2012).

### PARTICIPANTS

Thirteen AD participants aged 2-5 years (*M* = 43.16 months, SD = 11.71; 8 male, 5 female) with a diagnosis of an autism spectrum disorder (Autistic Disorder: *n* = 12, PDD-NOS: *n* = 1) were recruited from early intervention centers, medical and mental health clinics and by referral from local providers. Diagnoses were confirmed with Module 1 (*n* = 12) or 2 (*n* = 1) of the Autism Diagnostic Observation Schedule (Lord et al., 2000) using revised diagnostic algorithms (Gotham et al., 2007). Five additional children with ASD participated in the experiment, but were excluded from analysis because we were unable to calibrate them to the eye tracking system.

Eleven LD participants (*M* = 38.10 months, SD = 11.73; 9 male, 2 female) were recruited as age matched controls. LD participants were recruited through early intervention centers and by referral from local providers. LD participants had a diagnosis of expressive (*n* = 2) or mixed expressive/receptive language delay (*n* = 9). Delays in language were confirmed with the Preschool Language Scales (at least -1 SD; Zimmerman et al., 2002) or MacArthur Bates Communicative Development Inventories Words and Gestures or Words and Sentences (at least -6 months age equivalent) (Fenson et al., 2007). Eight additional LD participants were recruited but excluded from analysis because of poor calibration to the eye tracker (*n* = 5), cognitive and language testing were never completed (*n* = 2) or because parents reported a first degree relative with an ASD (*n* = 1).

Fourteen TD participants (*M* = 16.39 months, SD = 6.38; 10 male, 4 female) were recruited from an established research database of children born in the state as language matched controls. An additional eight TD infants were recruited but excluded from analysis because cognitive and language testing were never completed (*n* = 2), language skills were too high to match with AD participants (>1.5 SD above average) (*n* = 2), or because of poor calibration to the eye tracker (*n* = 4). Cognitive and language testing results are provided in **Table 1**.

**Table 1:** Test scores for autistic (AD) language delayed (LD) and typically developing (TD) groups.

	AD (*n* = 13)	D (*n* = 11)	TD (*n* = 14)
Age	43.16 (11.71)	38.10 (11.73)	16.39 (6.38)**
Cognitive	69.77 (11.26)	87.00 (10.70)**	103.57 (8.64)**
**PLS**
Expressive raw	24.85 (9.25)	32.18 (10.38)	25.79 (8.29)
Receptive raw	23.00 (10.72)	31.82 (9.35)^†^	22.93 (7.36)
Expressive standard	61.15 (10.69)	79.00 (11.94)**	107.57 (8.71)**
Receptive standard	56.46 (13.67)	80.09 (13.38)**	101.79 (12.94)**
CDI words	129.54 (212.64)	250.73 (210.37)	87.29 (160.79)
**ADOS**
Social	18.15 (2.94)	–	–
Repetitive	3.62 (1.94)	–	–
Score	21.69 (4.13)	–	–
Severity	8.54 (1.20)	–	–

Cognitive scores are standard scores from the Bayley Scales of Infant Development (AD: *n* = *8*; LD: *n* = 5; TD: *n* = 14) or the Wechsler Preschool and Primary Scale of Intelligence-III (AD: *n* = 5; LD: *n* = 6). PLS, Preschool language scales; CDI, MacArthur Bates Communicative Development Inventory is the raw number of words produced as reported on the Words and Gestures (AD: *n* = 9; LD: *n* = 3; TD: *n* = 12) or Words and Sentences form (AD: *n* = 4; LD: *n* = 8; TD: *n* = 2); ADOS, Autism Diagnostic Observation Schedule. ^†^indicates *p* < 0.10, **p < 0.01 in the contrast with AD participants.

### SCREENING VISIT

#### Parental surveys

Parents completed surveys regarding their child’s developmental history and demographic information. Both language-delayed and typical controls were screened for social impairment with parental report measures. Depending on the age of the child, we used the Communication and Symbolic Behavior Scales (CSBS) (Wetherby et al., 2002), the Modified Checklist for Autism in Toddlers (M-CHAT) (Robins et al., 2001), or the Social Communication Questionnaire (Rutter et al., 2003).

#### Cognitive and language tests

All participants underwent cognitive and language testing administered by trained staff and supervised by a licensed clinical psychologist. Cognitive tests were either the Bayley Scales of Infant Development, third Edition (Bayley, 2005), or the Wechsler Preschool and Primary Scale of Intelligence-III (Wechsler, 2002), depending on the age and verbal ability of the child. All participants were tested using the Preschool Language Scales (PLS) – forth Edition (Zimmerman et al., 2002). If cognitive or language testing had been completed by a licensed professional within the previous 6 months, we used the child’s existing scores. In one case, this resulted in scores obtained using the fifth Edition of the PLS.

### EXPERIMENTAL VISIT

#### Stimuli

Four familiar objects and twelve novel objects, matched on size, were used to create the stimuli. Each child saw the same eight trials in the same order: two training trials followed by six test trials. Each trial consisted of a familiarization and test phase. During familiarization, children saw videos of a woman sitting at a table with two objects in front of her. Following an animated greeting to signal communicative relevance (Senju and Csibra, 2008), the woman picked up one of the objects (hereafter, the target object) and began labeling it. The speaker manipulated the object while labeling. We chose this design because prior work has demonstrated that this is a context in which autistic children succeed at identifying a target referent (Parish-Morris et al., 2007). It is important to note that this manipulation created a critical difference between these stimuli and the “dyadic bid” stimuli used by Chawarska et al. (2012). While the stimuli were similar in that the speaker directed her attention towards the child while speaking, the motion of the objects as she labeled them added a highly salient nonsocial component to the stimuli, making this design more similar to the “moving toy” condition in the Chawarska et al. (2012) study.

**FIGURE 1:**
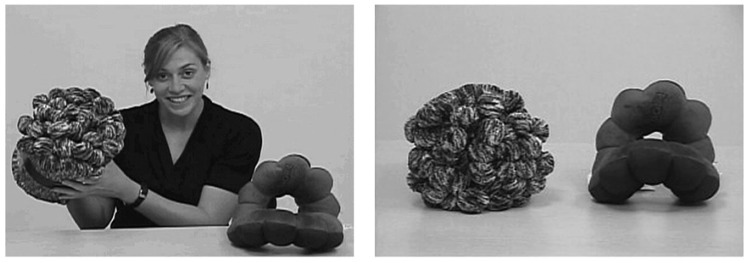
**Screen capture from the preliminary experiment.** During familiarization (left), the speaker labeled the target object (e.g., “Hey, it’s a toma!”) three times. She then picked up and identified the distracter object without assigning it a label (e.g., “Look at this.”). At test, children were told to “Look at the toma!” and an image of the objects appeared (right).

In the current design, as the speaker moved the objects, she labeled the target object three times (e.g., “Look, it’s a toma! Wow, what a great toma. Do you see the toma?”). Each time the speaker labeled the object, she shifted her gaze toward it, returning gaze to the camera after each label. She then picked up the other object (hereafter, the distracter object) and described it but did not provide a label (e.g., “Wow, look at this. Do you see this thing? It’s really something.”). When alluding to the distracter object, the speaker shifted her gaze between the object and the camera as she had done for the target object. The familiarization portion of the trial lasted 28 s. **Figure 1** shows a captured image from the familiarization and test phases. Target location and order of description were counterbalanced across trials.

After familiarization, an abstract image was displayed on screen while the test instructions were presented (e.g., “Look at the toma!”). The target and distracter objects then reappeared on the screen (the speaker was not in view for this test phase of the trials). The test phase of each trial ended once the child had successfully oriented to the target object and fixated on that object for 350 ms (or when 10 s had passed). This fixation time frame was based on the time it took children in pilot testing to shift their attention away from the target object after their first successful fixation on the target object. On training trials (the first two trials) children received positive feedback in the form of a happy face on screen while they heard “Yes!” or “Great!” once they had successfully oriented to the target familiar object during the test phase. On half the trials, the location of the objects was reversed between familiarization and test to ensure that the children were identifying the object rather than the location.

#### Eye tracking

Eye tracking was completed on a SensoMotoric Instruments (SMI) RED500 Remote Eye Tracking system (SensoMotoric Instruments, Inc., Boston, MA, USA). This system includes a remote-controlled infrared eye camera with automatic eye and head tracker. Tracking relies on a binocular image of the pupil and corneal reflection collected at a rate of 60 Hz with spatial resolution of 0.03° and gaze position accuracy of 0.4°. Blink recovery time is at maximum 4 ms and tracking recovery time for excessive movement is at maximum 90 ms.

An experimenter was seated at a computer adjacent to the display monitor, but hidden from view with a dark curtain. Calibration and stimulus presentation were displayed using the Experiment Center software provided by SMI. A two point calibration was used. This involved presentation of an animated image designed to attract the infant’s attention to fixation points at the top left and lower right corners of the screen. Calibration was then verified with a four point display of animated objects. Average deviation from calibration in the *X* plane was 1.03° (SD = 0.66) and in *Y*, 1.04° (SD = 0.61). Deviation from calibration did not differ significantly between groups, X: *F*(2,35) = 0.10, *p* = 0.90; Y: *F*(2,35) = 0.07, *p* = 0.94.

Stimuli (1600 × 1050 pixels) were presented on a 19″ (48.26 cm) computer monitor. Children were seated approximately 70 cm from the display monitor. At this distance, the woman’s face was 7 cm × 10 cm (eyes: 5 cm × 2.5 cm, mouth: 2.5 cm × 5 cm). The objects ranged from 10 to 15 cm^2^ during familiarization and 40–90 cm^2^ during test.

#### Procedure

Children sat on a caretaker’s lap or on a chair by themselves in front of the eye tracker and display screen. Each session began with the calibration described above. Once the child was successfully calibrated, testing began. At the start of each trial, an animated image was used to capture the child’s attention. Once the child had oriented to the screen, presentation of the first trial began. The procedure lasted between 5 and 10 min.

#### Analysis

**FIGURE 2:**
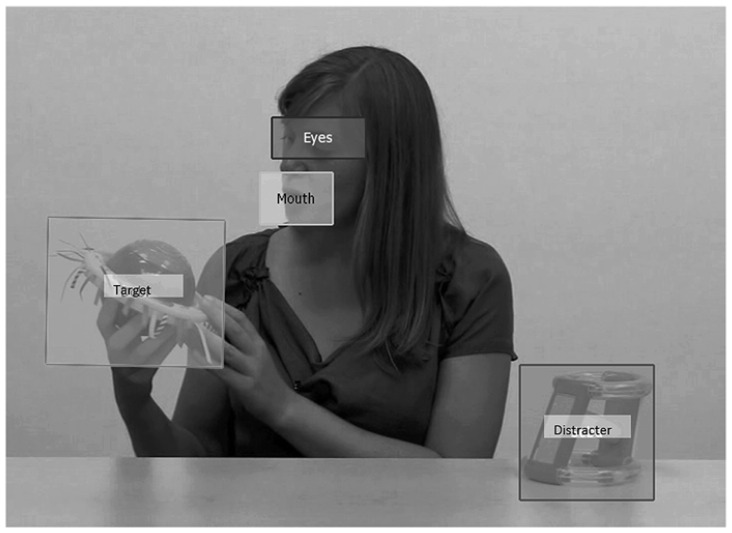
**Screen capture from familiarization trial with regions of interest (eyes, mouth, target, distracter)**.

Eye tracking data was preprocessed using SMI BeGaze native software (SensoMotoric Instruments, Inc., Boston, MA, USA). Fixations were defined as at least 100 ms spent fixating a 100 pixel area. We defined four dynamic regions of interest, frame by frame, for the familiarization trials: eyes, mouth, target, and distracter. **Figure 2** shows a screen capture from a familiarization trial including the defined regions of interest. For test trials, there were two regions of interest: target and distracter. Time spent fixating the eyes, mouth, target, and distracter were calculated as the percentage of tracked trial time (maximum 28 s) that each participant spent looking at those specific regions of interest. Speed of target recognition was calculated as time from the start of the test trial (when the objects reappeared after the instruction to “Look at the [target]” was given) until the participant had fixated the target object for at least 350 ms. As described above, this value was chosen based on time it took children in pilot testing to shift attention away from the target object after successfully fixating that location.

Previous work has demonstrated that typically developing infants gain speed in shifting attention to a target object over the course of development (Fernald et al., 1998) and that speed of word recognition is related to vocabulary size and grammatical development across the second year (Fernald et al., 2006) and into childhood (Marchman and Fernald, 2008). Fernald and colleagues used reaction time as a measure of recognition of familiar words (Fernald et al., 2008). Here we used latency of looks to a target after a single familiarization trial. This method necessitated the use of fewer trials than in Fernald’s approach (Fernald et al., 2008). This meant that there were not enough trials to allow for restriction of analysis to those on which a child first looked to the distracter, as was done in Fernald’s studies (Fernald et al., 2008). Consistent with Fernald’s approach, first looks in the current experiment tended to be evenly distributed between the target and distracter objects as target location was randomized across test trials (51.07%, of first looks were to the target, SD = 25.12). We therefore used latency rather than proportion of correct first looks because it more accurately reflects an active search for the target object.

## RESULTS

### ATTENTIONAL DISTRIBUTION ACROSS GROUPS

Preliminary analyses explored group differences in time spent fixating the stimuli overall, proportion of tracked time spent fixating the speaker’s mouth, eyes, target, and distracter objects, and speed of target recognition at test. On average, participants watched 62% (17.29 s) of the 28 s long familiarization stimuli (AD: *M* = 54.21%, SD = 19.78; LD: *M* = 66.22%, SD = 17.06; TD: *M* = 65.23%, SD = 16.73). All participants spent the majority of the familiarization trials focused on the objects (AD: *M* = 68.09%, SD = 17.17; LD: *M* = 67.38%, SD = 11.00; TD: *M* = 39.99%, SD = 12.56). That time was evenly divided between the target and distracter objects in all groups (AD Target: *M* = 30.96%, SD = 7.65; AD Distracter: *M* = 30.97%, SD = 7.04; LD Target: *M* = 34.19%, SD = 6.19; LD Distracter: *M* = 33.19%, SD = 7.69; TD Target: *M* = 34.33%, SD = 9.59; TD Distracter: *M* = 33.76%, SD = 9.01). Attention to the eyes (AD: *M* = 3.32%, SD = 4.97; LD: *M* = 4.82%, SD = 4.95; TD: *M* = 4.00%, SD = 5.51) and mouth (AD: *M* = 5.77%, SD = 4.85; LD: *M* = 3.34%, SD = 3.18; TD: *M* = 6.54%, SD = 6.72) were also relatively consistent across groups. Analysis of variance (ANOVA) revealed no significant effects of group for the distribution of attention to the stimuli overall *F*(2,35) = 1.76, ns, η^2^ = 0.09, attention to the mouth, *F*(2,35) = 1.19, ns, η^2^ = 0.05, eyes, *F*(2,35) = 0.25, ns, η^2^ = 0.01, target, *F*(2,35) = 0.76, ns, η^2^ = 0.05, or distracter object, *F* (2,35) = 0.46, ns, η^2^ = 0.02.

### PERFORMANCE AT TEST

Word learning within the task was quantified as time from the start of the test trial [when the objects reappeared after the instruction to “Look at the (target)”] until the participant fixated the target object for at least 100 ms. Autistic participants were slower on average (*M* = 2.89 s, SD = 3.49) than language delayed participants (*M* = 2.01 s, SD = 1.99) or typically developing participants (*M* = 1.69 s, SD = 9.94) in fixating the target object, though ANOVA revealed no significant effect of group, *F*(2,34) = 0.30, ns, η^2^ = 0.02.Though, as mentioned above, first looks were evenly distributed between the target and distracter objects, when participants’ first looks were to the target, that look was longer than if they had first fixated the distracter object. This suggests that children were actively searching for the target object (or remaining there if they were already attending to the target). This pattern was consistent within each group (AD: Target *M* = 218.17 ms, SD = 78.47, Distracter *M* = 185.86, SD = 137.92; LD: Target *M* = 220.67 ms, SD = 70.34, Distracter *M* = 141.98, SD = 63.64; TD: Target *M* = 201.00 ms, SD = 75.41, Distracter *M* = 170.89, SD = 68.27 but only reached significance across groups (paired sample *t*-test: *t*(31) = 2.56, *p* < 0.05).

### LANGUAGE LEARNING AND ATTENTIONAL DISTRIBUTION

We next asked whether attention during a word-learning interaction predicts language abilities on standardized measures of language development and within the word learning task. To that end, we ran a linear regression with group as a categorical measure to explore the predictive value of attention to the speaker’s eyes, mouth, and target object on PLS scores (composite raw scores) and latency to the target object at test as well as the interactions between group and these measures. The overall model for PLS composite scores, which included cognitive scores as a covariate, was significant, *F*(12,26) = 2.75, *p* < 0.05, *R*^2^ = 0.56. The main effect of attention to the target was significant β = 0.51, *p* < 0.01, as were the effects of group for AD vs. TD, β = 2.27, *p* < 0.05 and AD vs. LD, β = 2.62, *p* < 0.05. The interactions between attention to the target for AD vs. TD was significant, β = 1.93, *p* < 0.05 as was the interaction for attention to the mouth for AD vs. LD, β = -2.84, *p* < 0.01. While regressions within groups are justified only for significant interactions, the full set of results is reported below for exploratory purposes.

Follow up regressions within groups, factoring out cognitive scores, were significant for the AD, *F*(4,8) = 4.23, *p* < 0.05, *R*^2^ = 0.68, and TD, *F*(4,10) = 4.01, *p* < 0.05, *R*^2^ = 0.62, groups. For the autistic participants, both attention to the target and attention to the speaker’s mouth predicted higher language scores on the PLS (See **Table 2**). The regression was not significant for the LD participants, *F*(4,6) = 0.37, *ns*, *R*^2^ = 0.20, and no significant predictors emerged.

**Table 2:** Linear regression beta weights and significance for Preschool Language Scale composite raw scores within groups.

Predictors	AD*	LD	TD*
Cognitive scores	0.24	0.02	0.21
Attention to the target	1.22**	-0.46	-0.18
Attention to the mouth	0.95*	-0.12	0.72**
Attention to the eyes	0.10	0.21	-0.14

Linear regressions for AD: *F*(4,8) = 4.23, *p* < 0.05, *R*^2^ = 0.52 and TD, *F*(4,10) = 4.01, *p* < 0.05, *R*^2^ = 0.46, LD: *F*(4,6) = 0.37, *ns*, *R*^2^ = 0.34. Cognitive scores were based on the WPPSI or Bayley scores depending on the child‘s age. Attention to the target, mouth, and speaker were calculated at proportions of tracked trial time spent fixating each region of interest. *p < 0.05, **p < 0.01.

When age was added to the overall model it was again significant, *F*(13,25) = 10.86, *p* < 0.01, *R*^2^ = 0.85. In addition to the significant effects of the covariates age, β = 0.99, *p* < 0.01 and cognitive scores, β = 0.44, *p* < 0.05, the main effect of attention to the target was significant β = 0.44, *p* < 0.01, as were the effects of group for AD vs TD, β = 2.36, *p* < 0.01 and AD vs. LD, β = 2.00, *p* < 0.01. Also, the interactions between attention to the target for AD vs. TD, β = 1.51, *p* < 0.01, and for attention to the mouth for AD vs. LD, β = 0.63, *p* < 0.01, were significant. Again, though regressions within groups are justified only for significant interactions, we report the full set of results for exploratory purposes.

When age was added to the within group regressions all three were significant, AD: *F*(5,7) = 8.72, *p* < 0.05, *R*^2^ = 0.86, TD: *F*(5,9) = 17.67, *p* < 0.01, *R*^2^ = 0.91, LD: *F*(5,5) = 17.10 *p* < 0.01, *R*^2^ = 0.95. Age was a significant predictor of PLS scores for each group. When controlling for the effects of age, attention to the mouth and target object remained significant predictors of PLS scores for the autistic participants, but attention to the mouth was no longer a significant predictor of PLS scores for the typically developing participants (See **Table 3**). When controlling for age, attention to the mouth predicted *lower* language scores among language delayed participants.

**Table 3:** Linear regression beta weights and significance for Preschool Language Scale composite raw scores within groups factoring out cognitive scores and age.

Predictors	AD**	LD**	TD*
Age	0.57*	0.90**	0.80**
Cognitive scores	0.49	0.33	0.09
Attention to the target	0.97**	0.22	0.10
Attention to the mouth	0.62*	-0.41*	0.21
Attention to the eyes	0.35	0.04	0.09

Linear regressions for AD: *F*(5,7) = 8.72, *p* < 0.05, *R*^2^ = 0.86, TD: *F*(5,9) = 17.67, *p* < 0.01, *R*^2^ = 0.91, LD: *F*(5,5) = 17.10 *p* < 0.01, *R*^2^ = 0.95. Cognitive scores were based on the WPPSI or Bayley scores depending on the child‘s age. Attention to the target, mouth and speaker were calculated at proportions of tracked trial time spent fixating each region of interest. *p < 0.05, **p < 0.01.

Overall attention to the target, mouth and eyes did not reliably predict faster response to the target during the test phase of the experiment overall, *F*(13,26) = 0.42, *ns, R*^2^ = 0.19, or for any of the groups, AD: *F*(5,7) = 1.06, ns, *R*^2^ = 0.43, TD: *F*(5,7) = 1.03, ns, *R*^2^ = 0.42, LD: *F*(5,5) = 0.11 ns, *R*^2^ = 0.10. However, for exploratory purposes, we restricted the analysis to key portions of the trials that would be informative for learning the novel word. For gaze following, we used attention to the target relative to both objects while the speaker was manipulating the object (time spent fixating the target/time spent fixating the target and distracter objects while the speaker was manipulating the target object). For attention to the face, we used attention to the eyes and mouth while the speaker was labeling those objects (proportion of tracked time spent fixating the eyes or mouth averaged within each participant across the three repetitions of the novel label). We again factored out age and cognitive scores and the resulting regression was significant for autistic participants AD: *F*(5,7) = 4.39, *p* < 0.05, *R*^2^ = 0.69. As with the PLS scores, attention to the mouth emerged as a significant predictor of latency to the target among the autistic participants (see **Table 4**) attention to the eyes also predicted latency to the target object at test for autistic children. The regression models for TD and LD participants were not significant, and no significant predictors emerged for these groups, TD: *F*(5,7) = 1.31, ns, *R*^2^ = 0.50, LD: *F*(5,5) = 0.20, *ns*, *R*^2^ = 0.10.

**Table 4:** Linear regression beta weights and significance for latency to the target (where shorter times represent faster responses) within groups, factoring out cognitive scores and age.

Predictors	AD*	LD	TD
Age	-0.62*	0.03	0.01
Cognitive scores	-0.96**	-0.10	0.28
Attention to the target	-0.41	0.54	0.03
Attention to the mouth	-1.03**	0.53	0.20
Attention to the eyes	-0.68*	-0.10	0.50

Linear regression for AD: *F*(5,7) = 4.39, *p* < 0.05, *R*^2^ = 0.69, TD: *F*(5,7) = 1.31, *ns*, *R*^2^ = 0.50, LD: *F*(5,5) = 0.20, *ns*, *R*^2^ = 0.10. Cognitive scores were based on the WPPSI or Bayley scores depending on the child‘s age. Attention to the target object was the proportion of time spent fixating the target object/target + distracter objects while the speaker was manipulating the target objects. Attention to the mouth and eyes were calculated as the proportion of time spent fixating each region of interest/tracked time while the speaker was uttering the object label name (averaged across the three repetitions on each trial). *p < 0.05, **p < 0.01.

## DISCUSSION

This study explored the relations between attention to word learning stimuli, recognition of the newly learned word within the task, and standardized measures of language ability among autistic, language delayed, and typically developing children. Our results indicate that attention to the mouth is a significant predictor of standardized language scores among autistic and typically developing children. This effect holds when controlling for cognitive functioning and age among autistic children, but is mediated by age among typically developing children. Attention to the speaker’s mouth and eyes while she was providing the label for the object also predicted shorter latencies of first look to the target object among autistic children. This work adds to a growing body of evidence that attention to social stimuli, and specifically attention to a speaker’s mouth in the early word learning stages of development may be adaptive for language acquisition in typically developing and autistic children (Young et al., 2009; Chawarska et al., 2012). These preliminary findings raise the possibility that attending to a speaker’s face may enhance word learning among autistic children. While our method does not allow for a strong interpretation that word learning had occurred, it did allow for a measure of visual search for the target. Further, the results within the task were consistent with the results on a standardized measure of language development. Given the small sample for these novel findings, replication will be necessary to confirm that this pattern will hold in the broader population.

Contrary to our predictions and previous findings (Chawarska et al., 2012), attention to the stimuli did not differ between autistic children and controls on overall attention to the stimuli or distribution of attention within the social scene. This is likely due to the predominant focus on the objects in this experiment by all three groups (>60% of all tracked trial time was spent fixating the objects during the familiarization trials). The speaker in this experiment both picked up and manipulated the objects during each trial. This was done to simplify the task for autistic children to a situation in which their capacity to identify a target referent has been established (Parish-Morris et al., 2007). It also drew a great deal of attention to the objects. While Chawarska et al. (2012) did find differences between groups when there was a dyadic bid for interaction (that is, when the speaker in the video spoke to the child directly) there were not significant differences between the groups for the condition in which a toy on screen was moving. This is consistent with the current findings in which the objects were being held and manipulated for much of the familiarization trials.

Another potential reason for the lack of difference between groups in distribution of attention is that we matched subjects on language levels. Given that attention to faces has been demonstrated to shift as a function of both age and language ability (Lewkowicz and Hansen-Tift, 2012), it is possible that the matching on language ability obscured differences between the groups that emerged in previous studies of chronologically matched groups.

While attention to the mouth was shown to predict greater language skills and shorter latencies to target among autistic children, a different pattern of results was found for language delayed children. Among the LD group, when controlling for age, attention to the mouth was negatively correlated with language ability. Given that the language delayed children in this experiment were somewhat more advanced in their language skills than the autistic children, it is possible that they were beyond the level at which attention to the mouth is relevant for language learning. Though there is now a good deal of evidence demonstrating predominant focus on the mouth in infancy (Merin et al., 2007; Nakano et al., 2010; Frank et al., 2011; Lewkowicz and Hansen-Tift, 2012; Oakes and Ellis, 2012), it is not yet clear when attention shifts back to a more adult-like focus on a speaker’s eyes. Some have shown a shift as early as 12 months (Lewkowicz and Hansen-Tift, 2012), while others find focus on the mouth through early childhood (Nakano et al., 2010).

Lewkowicz and Hansen-Tift (2012) demonstrated that novel linguistic content serves to maintain infants’ attention on the mouth at older ages. The typically developing infants with the highest PLS scores (on par with those in the LD group) were most likely to focus attention on the mouth in this task. In light of this, we argue that in this novel word-learning context, even toddlers with verbal abilities above the autistic and typically developing participants could have benefited from focusing on the mouth. The fact that the language delayed participants who focused on the speaker’s mouth had lower scores on the PLS suggests that different mechanisms may drive connections between social attention and language development among autistic children and language delayed participants without autism.

The autistic participants in this group were quite young. Thus, we cannot be certain about the cognitive or linguistic profile these children will go on to develop (Tager-Flusberg, 2006). In this regard, the group of children with autism was likely to have been comprised of some children who will go on to have positive language outcomes and others who will go on to have poor language outcomes. Prior research has shown that joint attention abilities are predictive of language in children with autism (e.g., Mundy et al., 1990), and has motivated interventions that target joint attention as a way to support improved language outcomes (Kasari et al., 2006). Continued longitudinal research examining attentional distribution to faces and objects specifically in word learning contexts has the potential to help refine early interventions targeting language outcomes in this population. The results of this study indicate that patterns of visual attention in word-learning contexts may reflect processes of language development that manifest differently in children with autism as compared to children with language delays. Understanding the relationship between visual attention patterns and language learning has implications for studying the emergence of linguistic capacities in infants and toddlers with autism, and may help to guide and refine early interventions targeting word learning in children with autism and non-autistic children with language delays.

## Conflict of Interest Statement

The authors declare that the research was conducted in the absence of any commercial or financial relationships that could be construed as a potential conflict of interest.
